# Validity and reliability of a multiple-group measurement scale for interprofessional collaboration

**DOI:** 10.1186/1472-6963-10-83

**Published:** 2010-03-30

**Authors:** Chris Kenaszchuk, Scott Reeves, David Nicholas, Merrick Zwarenstein

**Affiliations:** 1 Keenan Research Centre in the Li Ka Shing Knowledge Institute, St. Michael's Hospital, Toronto, Canada; 2 Wilson Centre for Research in Education, University Health Network, Toronto, Canada; 3 Department of Psychiatry, University of Toronto, Toronto, Canada; 4 Faculty of Social Work, University of Calgary (Edmonton Division), Edmonton, Canada; 5 Research Institute, The Hospital for Sick Children, Toronto, Canada; 6 Sunnybrook Research Institute, Sunnybrook Health Sciences Centre, Toronto, Canada; 7 Department of Health Policy, Management and Evaluation, University of Toronto, Toronto, Canada; 8 Institute for Clinical Evaluative Sciences, Toronto, Canada

## Abstract

**Background:**

Many measurement scales for interprofessional collaboration are developed for one health professional group, typically nurses. Evaluating interprofessional collaborative relationships can benefit from employing a measurement scale suitable for multiple health provider groups, including physicians and other health professionals. To this end, the paper begins development of a new interprofessional collaboration measurement scale designed for use with nurses, physicians, and other professionals practicing in contemporary acute care settings. The paper investigates validity and reliability of data from nurses evaluating interprofessional collaboration of physicians and shows initial results for other rater/target combinations.

**Methods:**

Items from a published scale originally designed for nurses were adapted to a round robin proxy report format appropriate for multiple health provider groups. Registered nurses, physicians, and allied health professionals practicing in inpatient wards/services of 15 community and academic hospitals in Toronto, Canada completed the adapted scale. Exploratory and confirmatory factor analysis of responses to the adapted scale examined dimensionality, construct and concurrent validity, and reliability of nurses' response data. Correlations between the adapted scale, the nurse-physician relations subscale of the Nursing Work Index, and the Attitudes Toward Health Care Teams Scale were calculated. Differences of mean scores on the Nursing Work Index and the interprofessional collaboration scale were compared between hospitals.

**Results:**

Exploratory factor analysis revealed 3 factors in the adapted interprofessional collaboration scale - labeled Communication, Accommodation, and Isolation - which were subsequently corroborated by confirmatory factor analysis. Nurses' scale responses about physician collaboration had convergent, discriminant, and concurrent validity, and acceptable reliability.

**Conclusion:**

The new scale is suitable for use with nurses assessing physicians. The scale may yield valid and reliable data from physicians and others, but measurement equivalence and other properties of the scale should be investigated before it is used with multiple health professional groups.

## Background

Interprofessional collaboration (IPC) occurs when individuals from different health professions communicate and make decisions about a patient's health care based on shared knowledge and skills [1]. Collaborative practice among health care providers is coming to be viewed as a key component of strengthening Canada's health care system [2-4]. Interprofessional collaboration is expected to maximize health human resources, promote habits and customs leading to safer patient care, and increase satisfaction among patients and providers in Canada's health care institutions [5]. In the province of Ontario, an advisory committee to the Ministry of Health and Long-Term Care (MOHLTC) called for an evaluation framework to provide "evidence on the outcomes and benefits of interprofessional care," and for "sharing collective performance measures among peers" [5]. Clinicians trained in a varied array of health disciplines practice with nurses and physicians in many health care settings, including sites such as primary care and family medicine, emergent, intensive, and acute inpatient hospital care, and complex continuing care and rehabilitation [6]. A recognized need is emerging to broaden research and evaluation from the traditional focus on nurses and physicians to include other health professionals who have been regarded as allied with medicine and nursing. The Interprofessional Care Steering Committee emphasized this point by noting that, "in order to be inclusive and successful, all types of health caregivers must participate in implementing recommendations" presented in its action plan for interprofessionality [5,13].

The HealthForce Ontario program of the MOHLTC has funded projects to enhance the evidence base for IPC. We participated in a recent project funded by MOHLTC. A significant component of the project involved explicating some of our views of the status of IPC measurement. Accordingly, the present paper discusses a novel approach to measurement instrumentation. It presents initial psychometric results of a measurement scale designed for use with multiple health care provider groups in interprofessional collaboration research projects with measurement objectives.

### Rationale

The centerpiece of our approach was a commitment to obtain measurement survey coverage of all clinicians working in acute care wards using survey items constructed in a way that makes clinician respondents 'raters' of targeted clinician groups. In other behavioural research disciplines this structure has been called a *round robin *design [7,8]. To implement this structure we required an instrument designed for three groups of clinicians: physicians, nurses, and other regulated health care professionals. To make interprofessional comparisons, data were desired with survey items measured in a common scale. A measurement instrument was sought that had been developed for use with multiple groups of health professionals working in inpatient acute care hospital wards. Our search was guided in part by two reviews of instruments for nurse-physician collaboration [9,10]. In addition, we conducted a non-systematic examination of peer-reviewed literature in nursing, allied health care, and medicine journals, seeking IPC instruments developed for multiple rater/target groups and used in a round robin format. We could not find an instrument with these characteristics and we concluded that a suitable instrument was not available.

Two results of our review were influential. First, scales focused on relationships between two groups of health professionals, usually nurses and another group. Nurses and physicians should be central figures in measurement schemes but, in our opinion, not so dominant as to suppress considerations of professions allied with medicine and nursing, e.g., therapists, social workers. Other scales did not present common items to all of the health professional groups for whom the survey was intended.

Second, we were uncomfortable with decisions made (and not made) in the factor analytic approaches undergirding the development of scales presently in the literature. Many were developed using exploratory factor analysis (EFA). As implied by its name, EFA is exploratory, useful for initial investigation and development of scales to suggest factor structure dimensionality. EFA can be foundational for theoretical and hypothesis-driven refinements investigated in a confirmatory factor analysis (CFA) framework. However IPC researchers should recognize that EFA is primarily a data-driven method, and it appears as if the IPC measurement advances from EFA to CFA that one would expect (and hope) to see as construct validity is clarified have largely not occurred. As well, factor analysis experts have argued that EFA should not be used as a basis for final determination of a construct because EFA-based factor structures may not be reproducible in other data sets [11-13].

A guarded position towards EFA methods for IPC instruments was also motivated by a review [14] of factor analyses in a prominent nursing research journal. Some of the review's negative descriptions corresponded with our own reading of the IPC scale literature, from which three problems are highlighted. First, published factor analytic accounts do not report enough information about major decision points; reporting is highly selective. Second, sample sizes are at the low range of recommendations, which themselves are estimates that vary widely and appear with little support. Third, default software package options and suboptimal practices are often used, such as the well known Kaiser-Guttman rule for deciding the number of factors to retain, also known as the 'eigenvalues > 1' criterion [15-17]. This can be a problematic method in some circumstances [18,19] when one considers that the benefits of contemporary methods like parallel analysis and bootstrapping for factor retention have been shown.

The major problem with IPC instruments is that when responses to scale items are made by members of two clinical groups, and are assumed to be responses from *equivalent *groups, then a presumption of measurement equivalence/invariance (M-E/I) is imposed on the data. This means one is assuming that the same latent dimension is being assessed in both populations. Rather than assuming M-E/I, the issue should be viewed as an open hypothesis. Measurement invariance is a recent innovation in health services research [20], but rationale and methods for it have been presented in a variety of sociobehavioural research outlets [21-23]. In line with these arguments, our long-term goal is to develop a scale that is suitable for administration to key professional groups commonly working in acute care hospital wards - physicians, nurses, and allied health professionals. Ultimately this goal requires evidence that scale measurement invariance exists between multiple health professional groups. The evidence should include initial findings that an instrument is useful with at least one group.

Thus, we report here on the dimensionality and construct validity of a new scale for multiple-group measurement of IPC. Convergent, discriminant, and concurrent validity were assessed with a series of comparisons between the new IPC scale and two scales that have been used to measure facets of interprofessional collaboration, namely nurse-physician relationships and attitudes toward working in health care teams.

## Methods

Given limitations described above, data were collected to support factor analytic investigations of a multiple-group measurement scale. We considered the benefits of adapting an existing instrument against beginning scale development anew from a pool of candidate indicator items. Although it is commonly recommended, forming an item pool can be inefficient if it duplicates previous successful efforts and superfluous if less successful than previous efforts.

Nursing perspectives are central to a fully interprofessional approach. In particular, patient safety and quality of care in hospital settings are closely related to nursing practice, as are the sheer size of the nursing force, its relationship with medical practice (both maladaptive and complementary), and nursing shortages. Clearly, an instrument for interprofessional practice must be suitable for the nursing profession if it is to be useful for multiple groups. Lake's [24] review noted the Nurses' Opinion Questionnaire (NOQ) of the Ward Organisational Features Scales [25] as one of three leading measurement instruments for the nursing practice environment based on theoretical relevance. We adapted items appearing in several NOQ subscales to a new round robin format for use with multiple groups of health professionals. The NOQ's items tap important dimensions of IPC that are relevant for all acute care health professionals, such as discussion and movement of information among clinicians, cooperation, and conflict resolution. The relevant NOQ subscales were published with the labels Collaboration with Medical Staff, Collaboration with Other Health Care Professionals, and Cohesion Amongst Nurses [25].

### NOQ Adaptations

Subscales were adapted in two significant ways. First, some items in the nurse-physician NOQ subscale did not appear in the analogous subscales for relations between nurses and other healthcare professionals. For example, the item, "doctors are willing to discuss nursing issues," did not appear in the nurse-other subscale. Some items in the nurse-other subscale did not appear in the nurse-physician subscale, such as, "treatment carried out by other health care professionals often gives me cause for concern." This item was deleted. Items in the nurse cohesion subscale were intended for nurses, but two items were deemed important to include with nurse-physician and nurse-other assessments. These were, "important information is always passed on," and, "I feel nurses do not communicate with each other as well as they should." The second of these was revised to, "It is important to communicate well with [them]." We re-wrote items to render them presentable to members of three health professional groups with identical phrasings but for changes in the naming of the target group.

The second adaptation involved three items that appeared in the NOQ's nurse-physician and nurse-other subscales and addressed essentially-identical substantive target ideas. One item was written similarly in both subscales, but the other two were not. In the nurse-physician subscale one item read, "Doctors are usually willing to take into account the convenience of the nursing staff when planning their work." The analogous item in the nurse-other subscale was phrased as, "Other health care professionals ignore the convenience of the nursing staff when planning their work." One of these is phrased in a positive direction (the first, naming doctors) and the other in a negative direction. We standardized the item by writing it in the same direction (positive) for both target groups. Another item was also re-written for the same reason. For nurse-physician relationships it read, "medical staff cooperate with the way we organise nursing," and for nurse-other relationships the item was, "other health care professionals do not co-operate with the way we organise nursing."

The survey was constructed to elicit round robin ratings, meaning that items identified other clinical provider groups explicitly. Respondents self-identified their profession; we aggregated professions to higher-level groups. This was straightforward for nurses and physicians whose credentialing and licensure bestow common training within their professions. Members of other health professions - occupational and physical therapists, pharmacists, and social workers - were aggregated into a third group, *allied health staff*. This group encompasses a wide range of training backgrounds that may not be accurately represented by a catchall label but the many relevant core competencies that are common to allied health professionals, nurses, and physicians suggest that these professions have important, shared characteristics [6]. Aggregating data created six rater-target combinations. Naming the rating group first and the target group second, the rater-target dyads were: physicians-nurses, physicians-allied health staff, nurses-physicians, nurses-allied health staff, allied health staff-physicians, and allied health staff-nurses. It can be seen that the round robin design causes a group member to rate both other groups. In this respect respondents are proxy reporters on the collaboration behaviours of group targets. The round robin design also makes each group a target of two other groups.

Four response options were available for the items: strongly disagree (1), disagree (2), agree (3), and strongly agree (4). Five items were written in a negative direction; higher-numbered responses to these items - agreeing or strongly agreeing - represent an opinion that IPC was qualitatively worse. Numeric responses to negative items were recoded to align with positively-phrased items.

### Participants

The adapted IPC scale and other scales were administered to regulated health professionals in 15 community and teaching hospitals in Ontario, Canada as part of several independent IPC projects that occurred between 2006 and 2008. The projects had various objectives, such as intervention and evaluation, simulation, and organizational climate measurement. Survey completion was voluntary at all sites and was not linked either with occupational advancement or censure. Respondents received small incentives at some sites but not all, because compensation was determined within the context of independent research protocols and budgets. Survey responses were always confidential and de-identified from personal names and other identifying information. Approval for the study was granted by research ethics committees of the University of Toronto, the Humber Institute of Technology and Advanced Learning, Chatham-Kent Health Alliance, Children's Hospital of Western Ontario, the Hospital for Sick Children, Hotel Dieu Hospital, Kingston General Hospital, Lakeridge Health Corporation, London Health Sciences Centre, North York General Hospital, Rouge Valley Health System, St. Mary's General Hospital, the Scarborough Hospital, Sunnybrook Health Sciences Centre, Thunder Bay Regional Health Sciences Centre, Toronto East General Hospital, and Trillium Health Centre.

### Exploratory and confirmatory factor analysis

Because the properties of scales can change after being adapted [26], we performed factor analyses to investigate the properties of the instrument. Subscales were extracted from 14 items using 3 factor analysis steps and data combinations. In the first step exploratory factor analysis was conducted with data from 7 hospital sites (1 academic and 6 community hospitals) and responses from nurses evaluating physicians. Responses were treated as ordered categorical and were analyzed using the WLSMV (weighted least squares with mean- and variance-adjusted chi-square test statistic) estimator implemented in Mplus version 5.2. WLSMV is considered to be a strong estimation method for factor analysis with categorical data [27]. WLSMV provides statistical criteria to evaluate model fit in both exploratory and confirmatory modes, as well as conventional factor pattern coefficients ('loadings') and rotation methods. The main criteria for factor extraction were factor solutions based on eigenvalues > 1.0, model fit indices, and conceptual usefulness. All fit indices output by Mplus for WLSMV estimation are reported in the paper to demonstrate that model fit and evaluation were not enhanced by selective reporting of fit statistics. EFA solutions were rotated to enhance conceptual clarity on assumptions that the factors were either uncorrelated (orthogonal) or correlated (oblique). Varimax and promax rotations were examined; results from promax solutions were considered most useful and are reported. Varimax patten coefficients are not indices of model fit and were not reported. Models were evaluated by their ability to produce subscales that (a) suggested three or more items for retention on a subscale, (b) had salient item factor loadings, (c) displayed internal consistency of items, and (d) exhibited theoretical and conceptual clarity of factors and items for measuring interprofessional collaboration.

The second factor analytic step was a hybrid of exploratory and confirmatory modes: exploratory factor analysis within a confirmatory framework (E/CFA) [27,28]. E/CFA was employed as an intermediate step after EFA because it was not clear that moving to fully confirmatory mode was justifiable. E/CFA requires an analyst to pre-determine a number of factors and to estimate a model that loads all scale indicators on all factors. Based on exploratory results, indicator items are specified as anchor variables for factors on which they are hypothesized to load highest. Anchor items could be those that had the largest pattern coefficients in exploratory mode, for example. Factor variances are fixed to unity, factor covariances are freely estimated, non-anchor items are free to load on all factors and, unlike traditional EFA, indicator cross-loadings and residual covariances can be fixed to zero. This step was completed with data from nurses at 8 hospital corporations and sites different from those used in the EFA step (3 community and 5 academic hospitals, including 2 academic paediatric hospitals).

The third factor analytic step was fully confirmatory, and based on results obtained in the E/CFA analysis. Data from nurses at all hospital sites were combined for full CFA estimation. Results of fully confirmatory models were evaluated on goodness of fit, areas of localized strain, and interpretability of parameter estimates.

### Validity and other measures

Convergent and discriminant validity of the IPC scale were examined by incorporating data from measurement scales that are used frequently in research on interprofessional working relationships. These were the Collegial Nurse-Physician Relations Subscale of the Nursing Work Index (NWI-NPRS) [29] and the subscales of the Attitudes Toward Health Care Teams Scale (ATHCTS) [30]. Several versions of the NWI-NPRS have been published. There is considerable item overlap between different versions, and for consistency our survey protocol used the three items that were all reported in three specifications of the NPRS [24,29,31]. Items are shown in the appendix. Given that the new IPC scale was adapted from one designed to measure nurses' views of nursing relationships with physicians, a substantial correlation between nurse responses about physicians on the IPC scale and the NWI-NPRS was expected. The amount of correlation was taken as an indicator of the convergent validity of the IPC scale with the NWI-NPRS.

The ATHCTS consists of 3 subscales to measure self-reported facets of attitudes toward collective teamworking in health care groups. The subscales contain 21 items in total and have been named Attitudes Toward Team Value, Attitudes Toward Team Efficiency, and Attitudes Toward Shared Leadership/Physician Centrality in prior literature. Higher scores on the Shared Leadership subscale indicate greater endorsement of distributed decision-making and less belief that work of nurses and other professionals should be performed principally to support medical dominance in decision-making. ATHCTS items measure attitudes, beliefs and opinions more than actual working practices. Low or moderate correlations between the other-directed IPC and self-appraisal ATHCTS subscales were expected. Low correlations were taken to indicate discriminant validity of the IPC scale. As well, low correlations were expected between the NWI-NPRS and the ATHCTS subscales. Scale intercorrelations were estimated by confirmatory factor analysis.

Concurrent validity was examined by performing all pairwise hospital site comparisons of mean scale score differences for the NWI-NPRS and IPC subscales using nurses' ratings of physicians. Significantly different sitewise comparisons on the scales were examined. The extent of overlap in sitewise mean differences between the new IPC scale and the established NWI-NPRS should indicate whether the IPC scales have concurrent validity with a measure that is highly relevant for the nursing work environment like the NWI-NPRS. Individual survey respondents were conceptualized as being nested within hospitals and a multilevel model was estimated with one level-2 predictor (*hospital*) and no random effects. Written in composite form, the statistical model was:(1)

This is a means-as-outcomes model. Least squares means for hospitals were estimated and compared using PROC MIXED in SAS 9.1.

Hospital-level reliability of IPC measures was examined by two methods. First, nurses' scores on the IPC and NWI-NPRS scales were summed and aggregated to the hospital-site level. Polychoric correlations [32,33] were calculated between responses to the IPC subscales' indicators, and the average interitem correlation was examined for each hospital site. Second, interrater reliability of nurse responses across hospital sites was evaluated by the intraclass correlation [ICC (1, k)] using the SAS macro called %*INTRACC*, and the reported statistic labeled, "Shrout-Fleiss reliability: mean k scores." The ICC estimates stability of data at the hospital level. It indexes mean rater reliability of hospital-level data and is interpreted as the extent to which similar mean scores would be obtained if additional respondent samples were taken repeatedly from hospitals. It has been recommended that both average interitem correlation and ICC(1, k) should exceed .60 to justify group comparisons, i.e., between-hospital comparisons in this case [34].

## Results

### Construct validity: factor analysis

Exploratory factor analysis was conducted with raw data input to Mplus. Mplus computes a polychoric correlation matrix for ordered categorical data. The number of cases used in the analysis of nurses evaluating physicians was 144. The solution revealed 3 eigenvalues greater than 1.0 (6.17, 1.38, and 1.21), therefore solutions with 1, 2, and 3 factors were examined. For 1-factor and 2-factor solutions with promax rotation, χ^2 ^statistics for tests of model fit were 82.412, d.f. = 30, p < .001, and 50.928, d.f. = 26, p < .001. Root mean square error of approximation (RMSEA) and root mean square residual (RMSR) values were .112 (RMSEA) and .903 (RMSR) for a 1-factor solution and .082 (RMSEA) and .073 (RMSR) for a 2-factor solution. The 2-factor solution was preferred over the 1-factor solution based on model fit indices. Four factor pattern coefficients cross-loaded on the two factors (>.30) in the 2-factor solution, model fit was not deemed satisfactory, and we considered the 3-factor solution. The 3-factor model fit better than others (χ^2 ^test of model fit = 41.61, d.f. = 25, p = .027; RMSEA = .065; RMSR = .06). Simple structure [35] was obtained. Factor pattern coefficients for items are presented in Table 1. Most items loaded >.30 on 1 factor only and weakly or negatively on others; however 2 items had moderate cross-loadings on 2 factors (items 6 and 8). Item 14 did not load high enough (>.30) to justify placement on any factor. The 3-factor solution was retained and submitted to exploratory factor analysis within the confirmatory framework (E/CFA) [27].

**Table 1:** EFA 3-factor solution: Promax rotated loadings

Item	Factor 1	Factor 2	Factor 3
1. <We> have a good understanding with <them> about our respective responsibilities.	**0.501**	0.104	-0.093
2. <They> are usually willing to take into account the convenience of <us> when planning their work.	0.113	**0.820**	-0.069
3. *I feel that patient treatment and care are not adequately discussed between <us> and <them>.	**0.659**	-0.083	0.201
4. <We> and <they> share similar ideas about how to treat patients.	**0.614**	0.065	-0.110
5. <They> are willing to discuss <our> issues.	0.207	**0.524**	0.304
6. <They> cooperate with the way we organize <our> care.	0.074	***0.665***	***0.445***
7. <They> would be willing to cooperate with new <our> practices.	**0.616**	0.097	0.298
8. *The <they> do not usually ask for <our> opinions.	***0.400***	-0.017	***0.344***
9. <They> anticipate when <we> will need their help.	**0.511**	0.204	0.053
10. Important information is always passed on between <us> and <them>.	**0.649**	0.039	-0.097
11. *Disagreements with <them> often remain unresolved.	0.229	-0.120	**0.597**
12. *<They> think their work is more important than the work of <us>.	-0.108	0.186	**0.834**
13. *<They> would not be willing to discuss their new practices with <us>.	0.106	-0.041	**0.660**
14. It is important to communicate well with <them>.	-0.147	0.054	-0.003

Note: Terms in angle braces (< >) are replaced by professional group labels: physicians, nurses, or allied health staff.

* means the item is phrased negatively and responses were reverse-coded.

**Bold**: Candidate factor for item placement. *Italic: *Cross-loading item.

E/CFA requires selection of so-called 'anchor items' for estimating item loadings on each subscale. Anchor items were selected on the basis of results from the 3-factor EFA. The highest salient loadings from each factor were selected: for factor 1, item 3; for factor 2, item 2, and for factor 3, item 12. Other items were free to load on all factors, no cross-loadings were specified, and there were no correlated indicator errors. Factor variances were fixed to 1.0. Item 14 was dropped from this model because it showed no salient loading in EFA; its item-total correlation was low and negative in the EFA dataset (-.06, Pearson; average interitem polychoric correlation = -0.05 in the EFA dataset); and because an item such as this may have substantial negative impact on the accuracy of coefficient alpha reliability if it is not tau-equivalent with others [36,37]. Item 14 was, "It is important to communicate well with <them>." The number of cases used was 335.

The model's χ^2 ^value was 55.738, d.f. = 32, p = .006. CFI and TLI were .98 and .99 respectively; RMSEA was .047 and WRMR was .514. Completely standardized factor loadings and factor correlations are given in Table 2. Items are bolded to indicate likely factors for their placement. Within factors, items that were not selected for placement on a factor were never statistically significant (p < .05) or positive in direction. Comparing EFA results (Table 1) with E/CFA results (Table 2) shows that some items changed factors. Items 4 and 7 moved from EFA's factor 1 to E/CFA's factor 2. Item 8 cross-loaded weakly on factors 1 and 3 in EFA but loaded strongly on factor 3 in E/CFA. Item 6 had a moderate coefficient on factor 2 and slightly lower coefficient on factor 3 in EFA. In E/CFA analysis its loading on factor 2 was large. Item 11 loaded on factor 3 in EFA analysis but was not statistically different from zero on this factor in E/CFA, and indicated placement on factor 1 would be preferable. Some completely standardized estimates were > 1.0, which is permissible [38]. Based on E/CFA results, the theoretical and conceptual clarity of the factors and their indicators were reviewed, and labels applied. The constructs reflected theoretically supported and practically important constructs, which were named Communication, Accommodation, and Isolation. Constructs and items are in Table 3. Correlations between Communication and both other constructs were .86 and .90, suggesting that Communication may not be highly conceptually distinct from the others. R^2 ^values of indicators were examined and found to be distributed across acceptable ranges, between .35 and .71.

**Table 2:** E/CFA for a 3-factor model: Completely standardized coefficients

Factors/Items	Estimate	S.E.	Est./S.E.	Two-Tailed P-Value
*Factor 1*				
1	**0.999**	0.372	2.685	0.007
2	0.000	0.000	999.000	999.000
3	**0.608**	0.047	13.054	0.000
4	0.221	0.317	0.700	0.484
5	-0.581	0.363	-1.600	0.110
6	-0.862	0.502	-1.718	0.086
7	0.186	0.299	0.624	0.533
8	-0.060	0.246	-0.244	0.807
9	**0.798**	0.318	2.507	0.012
10	**1.748**	0.650	2.692	0.007
11	**0.728**	0.252	2.886	0.004
12	0.000	0.000	999.000	999.000
13	0.087	0.247	0.350	0.726
*Factor 2*				
1	0.208	0.222	0.938	0.348
2	**0.700**	0.035	20.226	0.000
3	0.000	0.000	999.000	999.000
4	**0.622**	0.174	3.577	0.000
5	**1.004**	0.206	4.869	0.000
6	**1.299**	0.304	4.276	0.000
7	**0.580**	0.158	3.673	0.000
8	0.163	0.146	1.121	0.262
9	0.110	0.196	0.562	0.574
10	-0.227	0.375	-0.607	0.544
11	0.005	0.158	0.030	0.976
12	0.000	0.000	999.000	999.000
13	0.051	0.145	0.354	0.723
*Factor 3*				
1	-0.665	0.306	-2.173	0.030
2	0.000	0.000	999.000	999.000
3	0.000	0.000	999.000	999.000
4	-0.333	0.231	-1.440	0.150
5	0.350	0.259	1.353	0.176
6	0.321	0.341	0.940	0.347
7	-0.076	0.220	-0.348	0.728
8	**0.717**	0.201	3.562	0.000
9	-0.320	0.240	-1.334	0.182
10	-0.983	0.474	-2.073	0.038
11	-0.064	0.196	-0.328	0.743
12	**0.724**	0.046	15.751	0.000
13	**0.545**	0.182	3.003	0.003

**Table 3:** Factor labels and items after E/CFA step

Num.	Factor label/text
	**Communication**
1	<We> have a good understanding with <them> about our respective responsibilities.
3	*I feel that patient treatment and care are not adequately discussed between <us> and <them>.
9	<They> anticipate when <we> will need their help.
10	Important information is always passed on between <us> and <them>.
11	*Disagreements with <them> often remain unresolved.
	**Accommodation**
2	<They> are usually willing to take into account the convenience of <us> when planning their work.
4	<We> and <they> share similar ideas about how to treat patients.
5	<They> are willing to discuss <our> issues.
6	<They> cooperate with the way we organize <our> care.
7	<They> would be willing to cooperate with new <our> practices.
	**Factor 3: Isolation**
8	*The <they> do not usually ask for <our> opinions.
12	*<They> think their work is more important than the work of <us>.
13	*<They> would not be willing to discuss their new practices with <us>.

* Reverse-coded

The final factor analytic step validated the E/CFA model by estimating a full confirmatory factor analysis model as suggested by E/CFA results. In the CFA model, nurse response data were used from all hospital sites available (n = 15), including the sites that contributed data to the EFA. Items were specified to load on their respective factors as shown in Table 3. No cross-loadings were specified and there were no correlated indicator errors. Factor variances were fixed to 1.0. The number of cases used was 479.

Model χ^2 ^was 147.98, d.f. = 44, p = .00. CFI and TLI values for the model were .94 and .98. RMSEA was .07 and WRMR was .84. These values are in acceptable ranges. According to recommendations by Hu and Bentler [39], model fit can be deemed acceptable if CFI and TLI are ≥ .95 and RMSEA ≤ .06. Browne and Cudeck [40] proposed that RMSEA values between .05 and .08 indicate fair model fit, and Bentler [41] suggested that CFI in the range of .90-.95 may indicate acceptable fit. A recent review of CFA fit statistics reported in more than 300 psychological reports published between 1998 and 2006 found mean CFI, TLI, and RMSEA values of .929, .904, and .064 [42]. WRMR has not been studied extensively but values less than .90 [28] and .95 [43] have been suggested as cutoff criteria for acceptable model fit for binary indicators.

Modification indices (M.I.) indicated several potential changes. The largest modification index suggested specifying item 10 ("important information is always passed on...") to load on factor 3 (Isolation) instead of factor 1 (Communication; M.I. = 19.4). While we felt this was theoretically reasonable, we did not change it. Modification indices also suggested moving items 6 and 7 ("they cooperate with the way we organize our care," and "they would be willing to cooperate with our new practices") from factor 2, Accommodation, to factor 1, Communication. These alternatives were rejected.

Completely standardized parameter estimates are given in Table 4. All coefficients were statistically significant (p < .05), larger than a frequently used standard for salience of .10 [44] and within ranges (0.3 to 1.0) reported in a review of standardized loadings commonly used in Monte Carlo studies [45]. Factor intercorrelations were .85 (Communication-Accommodation), .83 (Communication-Isolation), and .75 (Accommodation-Isolation), and were significantly different from zero. Although the correlation between Communication and Accommodation is high, we believe that the items defining the constructs are sufficiently unique to IPC measurement, and distinctly important enough to maintain as two separate constructs. R^2 ^values for the indicators were between .31 and .62. These values are within typical ranges for structural equation models [44] and we judged them adequate for a confirmatory model of the subscales.

**Table 4:** Full CFA model, full validation dataset, nurses→physicians: Completely standardized coefficients

Factors/Items	Estimate	S.E.	Est./S.E.	Two-Tailed P-Value
Communication				
1	0.58	0.05	12.94	0.00
3	0.64	0.04	15.63	0.00
9	0.63	0.04	17.97	0.00
10	0.61	0.03	17.82	0.00
11	0.67	0.04	17.86	0.00
Accommodation				
2	0.71	0.03	25.25	0.00
4	0.56	0.04	13.44	0.00
5	0.78	0.03	31.61	0.00
6	0.79	0.03	30.24	0.00
7	0.76	0.03	26.65	0.00
Isolation				
8	0.78	0.03	26.81	0.00
12	0.71	0.03	22.60	0.00
13	0.67	0.04	18.09	0.00

To take advantage of the round robin design of the IPC scale, the 3-factor model was estimated with all pairwise rater-target group combinations using data from all hospital sites. Examining fit indices and other results of these models is an informal approach to measurement equivalence of the scale for multiple professional groups. Building on results of nurses' assessments of physicians presented above, completely standardized parameter estimates from the CFA model fit to allied health professionals' responses about physicians are given in Table 5.

**Table 5:** Full CFA model, full validation dataset, allied→physicians: Completely standardized coefficients

Factors/Items	Estimate	S.E.	Est./S.E.	Two-Tailed P-Value
Communication				
1	0.46	0.06	8.17	0.00
3	0.70	0.05	14.11	0.00
9	0.52	0.06	9.12	0.00
10	0.63	0.05	14.02	0.00
11	0.62	0.05	12.94	0.00
Accommodation				
2	0.61	0.05	11.72	0.00
4	0.51	0.06	8.99	0.00
5	0.65	0.05	14.24	0.00
6	0.72	0.04	19.48	0.00
7	0.76	0.05	15.70	0.00
Isolation				
8	0.71	0.05	13.37	0.00
12	0.81	0.04	20.89	0.00
13	0.70	0.05	15.30	0.00

All item loadings on the constructs were significantly different from zero. Factor intercorrelations were .86 (Communication-Accommodation), .78 (Communication-Isolation), 2 and .77 (Accommodation-Isolation). The number of cases used in the analysis was 217. Model χ^2 ^was 79.109, d.f. = 27, p < .001. CFI and TLI were .92 and .95. RMSEA was .09 and WRMR was .98. This model fits the data reasonably well, however, modification indices indicated that model fit could be improved by moving item 4, "<we> and <they> share similar ideas about how to treat patients," from factor 2 (Accommodation), to factor 1 (Communication). Doing so improved model fit: model χ^2 ^decreased to 63.143 and RMSEA to .079. CFI and TLI increased to .95 and .96 respectively. The three pairs of factor correlations were all about .75.

Model fit results for all pairwise rater-target group combinations are shown in Table 6. Rows 4 and 5 of the table show results of nurse assessments made by physicians and allied health professionals. Both models fit the data about equally well with somewhat better fit suggested by allied health professionals' ratings of nurses than physicians' ratings of nurses. Results for models with allied health professionals as targets are in rows 6 and 7. The model for nurses' responses is marginally acceptable but is superior to the model for physicians' responses, which fits poorly.

**Table 6:** Model fit statistics for CFA models, by rater-target group combinations

Raters-targets	N	**Model χ**^**2**^	df	P	CFI	TLI	RMSEA	WRMR
N^1^→P	479	147.983	44	.00	.938	.976	.070	.841
A→P	217	79.109	27	.00	.925	.947	.094	.982
A→P^2^	217	63.143	27	.00	.948	.963	.079	.880
P→N	127	53.415	23	.00	.934	.943	.102	.913
A→N	215	82.850	27	.00	.947	.969	.098	.947
N→A	475	195.038	37	.00	.898	.967	.095	1.188
P→A	127	104.430	24	.00	.823	.904	.162	1.143

^1^N: Nurse; P: Physician; A: Allied Professional

^2^Model for moving item 4, "share similar ideas...", from Accommodation to Communication.

### Construct validity: convergent and discriminant validity

The IPC scales were developed from items in an instrument originally designed to measure nurses' work relationships with physicians and other professional health care staff. Many of these items were thought to resemble the essential aspects of nurse-physician relationships tapped by the NWI-NPRS. Therefore we hypothesized that the IPC scales would have large correlations with the NWI-NPRS among nurses. Both the IPC and NWI-NPRS scales tend towards measurement of behavioural aspects of interprofessional working instead of attitudinal aspects, and are other-directed, which means they should have low or moderate correlations with ATHCTS subscales. Factor correlations were estimated using data from all hospital sites. ATHCTS items, which had 6-category response options, were specified as categorical data for estimation in Mplus with the WLSMV estimator.

Table 7 shows factor correlations for nurses' responses about physicians on the IPC subscales, estimated from a 7-factor confirmatory model that included the 3-factor IPC scale targeting physicians, the NWI-NPRS items relating to physicians, and the 3-factor ATHCTS scale. Correlations between the NWI-NPRS and the 3 IPC factors of Communication, Accommodation, and Isolation were .80, .73, and .67 respectively, indicating some conceptual overlap between all IPC subscales and the NWI-NPRS. In contrast, the IPC subscale correlations with the ATHCTS subscales were considerably lower (between .2 and .4) or negative (-.28 and -.20). In similar fashion the correlations between NWI-NPRS and ATHCTS subscales were between .10 and .20, or -.25.

**Table 7:** Factor correlations: Nurses→Physicians

	Comm	Accom	Isol	NWI-NPRS	**TeamVal**^**+**^	**TeamEff**^**+**^	**SharedLdr**^**+**^
Comm							
Accom	0.85						
Isol	0.83	0.75					
NWI-NPRS	0.80	0.73	0.67				
TeamVal	0.31	0.19	0.19	0.17			
TeamEff	0.30	0.27	0.40	0.12^	0.75		
SharedLdr	-0.28	-0.20	0.01^	-0.25	0.01^	0.39	

^+ ^TeamVal = Team Value; TeamEff = Team Efficiency; SharedLdr = Shared Leadership

^ = n.s.; all others p < .05

Table 8 shows correlations for nurses' responses about allied health professionals for the same scales. Recall that the IPC scale had marginal fit according to model fit indices; results are shown to demonstrate the relationship of the IPC subscales to the NWI-NPRS scale. Correlations between IPC subscales that targeted allied professionals, and the NWI-NPRS scale (that targeted physicians), were low: .38, .36, and .25. These correlations corroborate the distinctiveness of the IPC subscales targeting allied professionals from the NWI-NPRS scale targeting physicians.

**Table 8:** Factor correlations: Nurses→Allied Professionals

	Comm	Accom	Isol	NWI-NPRS	TeamVal	TeamEff	SharedLdr
Comm							
Accom	0.81						
Isol	0.84	0.75					
NWI-NPRS	0.38	0.36	0.25				
TeamVal	0.27	0.24	0.19	0.17			
TeamEff	0.51	0.38	0.39	0.12^	0.75		
SharedLdr	-0.05^	-0.07^	0.01^	-0.25	0.02^	0.39	

^ = n.s.; all others p < .05

Table 9 gives correlations for allied professionals' scale responses about physicians, excluding NWI-NPRS items because they were not given to allied professionals. These correlations should be considered in comparison with Table 7. Among allied professionals the IPC subscales had large correlations resembling those seen in Table 7, where nurses' items targeted physicians. With allied professionals targeting physicians, the IPC subscales were distinct from the ATHCTS measures, as demonstrated by low to modest correlations, none greater than |.48|, and several not statistically significant.

**Table 9:** Factor correlations: Allied Professionals→Physicians

	Comm	Accom	Isol	TeamVal	TeamEff	SharedLdr
Comm						
Accom	0.86					
Isol	0.78	0.77				
TeamVal	0.17^	0.34	0.08^			
TeamEff	0.11^	0.15^	0.13^	0.71		
SharedLdr	-0.48	-0.46	-0.27	0.10^	0.16^	

^ = n.s.; all others p < .05

Correlations in Tables 10 and 11 relate to nurses as targets of items for physicians and allied professionals. Low correlations between IPC and ATHCTS subscales verify the conceptual distinction between the constructs.

**Table 10:** Factor correlations: Physicians→Nurses

	Comm	Accom	Isol	TeamVal	TeamEff	SharedLdr
Comm						
Accom	0.77					
Isol	0.66	0.66				
TeamVal	0.31	0.42	0.34			
TeamEff	0.35	0.40	0.21	0.89		
SharedLdr	-0.08^	-0.29	0.45	0.68	0.41	

^ = n.s.; all others p < .05

**Table 11:** Factor correlations: Allied Professionals→Nurses

	Comm	Accom	Isol	TeamVal	TeamEff	SharedLdr
Comm						
Accom	0.85					
Isol	0.83	0.73				
TeamVal	0.26	0.29	0.27			
TeamEff	0.36	0.26	0.48	0.71		
SharedLdr	-0.37	-0.31	-0.18^	0.09^	0.17^	

^ = n.s.; all others p < .05

Based on predictions and tabled results, the IPC subscales administered to nurse respondents targeting physicians showed moderate to high correlations with the NWI-NPRS, suggesting convergent validity of the IPC subscales. Nurses' responses to IPC scales targeting allied professionals were weakly correlated with NWI-NPRS responses targeting physicians. Both the IPC subscales and the NWI-NPRS scale had very low correlations with ATHCTS subscales, which is evidence of discriminant validity of the IPC subscales for distinguishing between targets - self and other.

The predicted patterns of large positive correlations between the IPC and NWI-NPRS scales - and small correlations between both instruments and the ATHCTS subscales - generalize across different rater groups of a common target group, namely, nurse and allied professional ratings of physicians, physician and allied professional ratings of nurses, and nurse ratings of allied professionals.

### Reliability of scales

Reliability statistics are presented in Table 12. Internal consistency reliability of the IPC factors was estimated with Raykov's composite reliability statistic, ρ [46]. Among nurses rating physicians at all sites, ρ values were .76, .85, and .76 (Communication, Accommodation, Isolation, respectively). Reliability was .92 for the NWI-NPRS. Among allied health professionals rating physicians, ρ values were .73, .79, and .79 (Communication, Accommodation, Isolation) and were .82, .88, and .72 for ratings of nurses. For physicians' IPC scale assessments of nurses, reliability was .80, .86, and .71 (Communication, Accommodation, Isolation).

**Table 12:** Reliability statistics for scales, by type and rater-target groups

Raters-Targets	Communication	Accommodation	Isolation	NWI-NPRS
*Composite reliability (individuals)*				
N→P	.76	.85	.76	.92
A→P	.73	.79	.79	
P→N	.80	.86	.71	
A→N	.82	.88	.72	
*Average interitem correlation (hospitals)*				
N→P^1^	.38	.59	.78	
A→P^1^	.03	.52	.63	
P→N^2^	.17	.47	.51	
A→N^1^	.58	.64	.68	
*ICC(1, k) (hospitals)*				
N→P	.99	.95	.97	.71
A→P	.95	.75	.94	
P→N	.87	.86	out of range	
A→N	.89	.50	.64	

^1^Polychoric. ^2^Pearson.

Hospital-level reliability was variable. For nurses' physician assessments, average interitem polychoric correlations for Communication and Isolation were .38 and .78. Reliability calculation for the Accommodation subscale employed Pearson correlations for 2 sites' data and polychoric correlations for others because polychoric calculation requires iteration [47] and a polychoric solution could not be attained. Average interitem correlation was .59. In other rater-target combinations, values ranged from very low to moderate for the Communication subscale and were closer to the recommended .60 value [34] for other subscales.

ICC(1, k) values were moderate to large for most rater-target dyads for most subscales. Physicians' ICC(1, k) value was out of range for Isolation due to lack of sufficient response variation between items.

### Concurrent validity: sitewise (hospital) comparisons of mean scale scores

The new IPC scales and the NWI-NPRS should have comparable abilities to detect statistically significant mean scale score differences between hospital sites. Accordingly, differences of least squares means of IPC and NWI-NPRS summated scores were tested across hospital sites. For the purpose of creating summated scale scores, cases with missing data in a scale item were deleted. At one hospital site the NWI-NPRS was administered to some nurses and not others; all responses from this site were used.

Pairwise hospital comparisons with statistically significant mean scale score differences are shown in Table 13 for nurses assessing physicians. These address concurrent validity between the IPC and NWI-NPRS. For further insight on measurement equivalence, results of allied professionals rating physicians are given in Table 14. The round robin aspect is incorporated with results of physician assessments of nurses in Table 15. Results are presented based on raw p-values and p-values adjusted for multiple comparisons by Tukey's honestly significant difference. As expected, fewer tests met the conventional criterion of p < .05 when p-values were adjusted for multiple comparisons.

**Table 13:** Hospital sites with significant mean differences on scales: Nurses rate physicians

Site lower												
**Site higher**	**1**	**2**	**3**	**4**	**5**	**6**	**7**	**8**	**10**	**11**	**12**	**15**

1												

2				N	N*, C, A, I							

3					N							

4					N, I							

5												

6				C	N, C, **I**							

7				A	N, C, A, I							

8					N, I							

10**	C		I	C, I	N,**C,**A,**I**			C, I		C, I		

11					N, I							

12	I		I	N, A, I	**N,**C, A,**I**	I		I		I		

15**	C, I		I	C, I	N, C, **I**	I		C, I		C, I		

N = NWI-NPRS; C = IPC Communication; A = IPC Accommodation; I = IPC Isolation

* Bold: unadjusted *and *adjusted p < .05

** Sites 9 and 13 not included (9, no NWI-NRPS; 13, no IPC responses)

**Table 14:** Hospital sites with significant mean differences on subscales: Allied professionals rate physicians

Site lower														
**Site higher**	**1**	**2**	**3**	**4**	**5**	**6**	**7**	**8**	**9**	**10**	**11**	**12**	**13**	**15**

1					I						I			A, I

2			A	A	A, I			A, I		I	A, I			A*,I

3														

4					I									I

5														

6			C		A, I			C			I			A, I

7			C		A, I						I			A, I

8					I									A, I

9			C		I						I			A, I

10			C											A

11														A

12			C		I						I			A, I

13			C		C, A, I			C			I			**A,**I

15**														

C = IPC Communication; A = IPC Accommodation; I = IPC Isolation

* Bold: unadjusted *and *adjusted p < .05

** Site 14 not included (incomplete data)

**Table 15:** Hospital sites with significant mean differences on subscales: Physicians rate nurses

Site lower													
**Site higher**	**1**	**2**	**3**	**4**	**5**	**6**	**7**	**8**	**9**	**11**	**12**	**13**	**15**

1													

2													

3													

4													

5													

6													

7													

8						A			I				

9					C	C, A							

11**	C, A			A	C, A	C, A		C, A	A				

12	C, A, I			A	C, A, I	C,**A**		C, A	C, A, I				

13	C*, A,I		A	C, A	**C, A,**I	**C, A,**I	C, A	C, A	C,**A,**I				

15**	A, I				A, I	A			A				

C = IPC Communication; A = IPC Accommodation; I = IPC Isolation

* Bold: unadjusted *and *adjusted p < .05

** Sites 10 and 14 not included (incomplete data)

Among nurses, two hospital sites were identified on the NWI-NPRS as having at least one significantly lower mean scale score than other sites on the basis of raw p-values: sites 4 and 5 (Table 13). As well, mean IPC scores at site 5 were lower than scores of most other sites, most frequently on the Isolation scale. In this respect there was substantial concordance between the NWI-NPRS and the IPC subscales. When there was a significant pairwise difference involving the NWI-NPRS, there was also a significant mean difference involving one or several IPC subscales. Analyses using adjusted p-values revealed 4 comparisons with significantly different IPC subscale scores that did not have a multiplicity-adjusted significant difference among NWI-NPRS scores; all involved site 5.

Sites 3, 5, 11, and 15 had patterns of low ratings of physicians when assessments were made by allied professionals (Table 14). Three sites (3, 11, 15) were not frequently identified by nurses in pairwise comparisons. Compared with nurses, allied professionals' ratings revealed more significant differences involving the Accommodation subscale than nurses, whereas nurse ratings were more likely to be lower on the Communication subscale.

For physician respondents targeting nurses with their assessments (Table 15), unadjusted p-value results were similar across IPC subscales. For example, sites 1,5, 6, and 9 were identified on every subscale with lower mean scores than at least one other site, usually sites 11, 12, and 13. Patterns were similar among adjusted p-value results: sites 1, 5, and 6 (or combinations) had lower mean scores than sites 12 or 13. Where there were significant pairwise differences on subscales for physician ratings, there was a tendency to observe a co-occurrence of the Communication and Accommodation subscales in a pairwise comparison.

Results compared across nurses and physicians showed both consistency and variation. Site 5 was frequently identified by both clinician groups as having lower mean scores than other sites, and site 12 as having higher scores. Among physician assessments of nurses, the prominent feature was the sites scoring higher (11-13), whereas for nurses rating physicians it was the lower scoring sites (4 and 5) which were most apparent.

## Discussion

This paper described initial development of a scale for multiple-group IPC measurement. We noted that measurement instruments for IPC have been conceptualized from a perspective of one or two clinical groups, typically nurses and another. As a result, there is little evidence that existing instruments have demonstrated measurement equivalence among three groups who often practice together - nurses, physicians, and allied health professionals. In noting this, we part ways with past work because we believe that it is important to begin measuring IPC among multiple clinical provider groups with a measurement scale that demonstrates meaningful measurement invariance.

An existing scale designed for nurses was adapted to a round robin format to place respondents in the position of being 'raters' of items' 'targets.' Construct validity was assessed by two factor analytic methods: factors extracted by exploratory methods were validated on alternate data in CFA frameworks. A CFA model without item cross-loadings or correlated residuals fitted to nurses' responses about physicians fit well according to fit indices, salience of item loadings on factors, and conceptual clarity, and fit moderately well when evaluated by residuals and item R^2 ^values.

Three key factors relevant to IPC were identified and labeled as Communication, Accommodation, and Isolation. By intention, one factor (Isolation) was permitted to be defined exclusively by negatively phrased items that existed in the original NOQ [25]. We made this allowance to acknowledge that negative aspects of interprofessional care may exist and should be measured appropriately. One of the essential findings of literature on the doctor-nurse game [48] was that nurses should not be openly critical of physicians. Hence, a measurement framework for an important aspect of IPC such as nurse-physician relations should accommodate this tendency. If a minor legacy of nurses protecting physicians from criticism continues to exist today, then it is possible that negative items must be employed in measurement studies because nurses could be reluctant to disagree with positive items if disagreement is their only option available to express negative opinions. Defining a factor based on negative items acknowledges that survey scales *convey *information as much as they elicit it [49,50]. Negative items put forth to respondents the researcher's awareness that nurse-physician relationships, and others, can be strained [51] and that the doctor-nurse game has not ended but continues to admit new players.

When fitted to responses of physicians targeting nurses and allied professionals targeting physicians, the fit of the models degraded slightly. For physicians the residual correlation matrix contained many large residuals, suggesting that more factors may be required. We did not pursue this possibility because we wished to guard against empirical and conceptual dilution of the factor structure.

Convergent and discriminant validity of the IPC subscales were examined by estimating factor correlations by CFA. Factors of the new IPC scale had correlations between .66 and .85. Correlations of this magnitude may raise doubt about the conceptual distinction of the factors; however some evidence suggests that when ambiguity exists, more factors should be extracted from data because over-factoring may have fewer disadvantages than under-factoring [52]. Moreover we were satisfied with the theoretical importance of the 3 factors, and their correlations. The NWI-NPRS demonstrated correlations with the IPC subscales ranging between .67 and .80. Both the NWI-NPRS and IPC subscales were weakly correlated with ATHCTS scales, thus demonstrating a distinction between an attitudinal self-appraisal scale and the other-directed bedside focus of the IPC scales and the NWI-NPRS. Similar correlation patterns were found for physicians' responses about nurses and responses to ATHCTS items, although correlations between IPC factors were lower among physicians than nurses.

Differences of mean scale scores from hospital sites were examined with the NWI-NPRS and IPC subscales. The NWI-NPRS was employed as a criterion on a hypothesis that the pattern of results obtained from a well-studied scale such as the NWI should be replicable to some degree by a new scale. This hypothesis was confirmed generally among nurses' assessments of physicians. The new IPC subscales distinguished many of the same sites as the NWI-NPRS with lower mean scores. In addition, the new scale identified several sites that were not distinguishable on the NWI-NPRS. This particular finding should be viewed as an advantage for the IPC scales, but it should not be interpreted as an indication of global superiority of the IPC subscales over the NWI-NPRS or the NWI in totality. Versions of the NWI may contain other scales that can measure essential concepts of the IPC scale in other ways; Aiken and Patrician's NWI-R contains a scale to measure autonomy, for example [29]. Additionally, our protocol employed an NWI-NPRS item pool that was relatively constricted compared to some others [31].

Nurses' and physicians' mutual ratings were not merely mirror images of one another. Where nurses rated physicians high (or low), physicians tended not to rate nurses similarly high (or low). In other words, ratings reciprocity was uncommon. This is reflective of recent quantitative reports on nurse-physician relationships which have shown that nurses' and physicians' opinions of each other's collaborative effectiveness are asymmetrical. A consistent body of studies has revealed that physicians have higher satisfaction with nurse-physician collaboration than nurses, and that physicians are less critical of nurses' collaboration efforts than nurses are of doctors' efforts [53-55]. Nurses have reported lower levels of communication openness with doctors [56] and lower quality of collaboration and communication with doctors than doctors of nurses [57,58]. Nurses are more likely to report problematic team- and communication-related behaviours that might endanger patient safety than either physicians or non-clinician managers [59]. This literature indicates that asymmetrical patterns of mutual nurse-physician assessments are normative, thus lending further support to the concurrent validity of the new IPC subscale.

### Limitations

First, the adapted scale is based on a nursing-centered questionnaire and no new items have been created or adapted from interviews from perspectives of a non-nursing health discipline. If perspectives of other disciplines are vital to measuring fully interprofessional collaboration, they may not be represented in the new scale. Second, the paper suggests but does not undertake the steps for investigating measurement equivalence. A reasonable analysis would investigate the following forms of measurement equivalence: (1) dimensional - the same number of common factors exist for all clinician-respondent groups; (2) configural - items load best on the same factors for all groups; (3) metric/pattern - the magnitude of item pattern coefficients is invariant across groups; and (4) strong factorial - invariant item thresholds (intercepts). Third, model fit statistics informally suggest that measurement equivalence may exist when nurses and physicians are item targets, but poor model fit when allied professionals were targets implies that the IPC scale may not be suitable for judgments of allied professionals considered as a homogeneous group. Future analyses should be conducted on disaggregated groups, such as physiotherapists. Finally, validity results would be strengthened by evidence that IPC as measured by the scale correlates with other important and relevant health outcomes indicators.

## Conclusion

The new IPC scale compares well with a known measure for nurse-physician teamwork. Consequently, it is useful for measuring nurse judgments of physician IPC. This is imperative because the sheer size of the nursing profession and its lockstep relationship with the field of medicine means that a scale intended for multiple groups must be suitable for nurses. Data from other dyadic group combinations should be interpreted cautiously until psychometric properties are explored. The scale is promising in other respects. It incorporates all major groups of health professions. It opens a possibility for other forms of validity investigation, such as multitrait-multimethod analyses [60]. The round robin design has two advantages: it ensures that raters and targets are identified clearly for respondents, researchers, and users of the measurement outcomes, and it promotes formal engagement with measurement equivalence and related issues of item bias and differential item functioning.

## Competing interests

The authors declare that they have no competing interests.

## Authors' contributions

CK conceived and designed the study, performed statistical analysis and interpreted results, and drafted the manuscript. SR contributed to study design and conception and manuscript drafts. DN participated in data acquisition, data analysis and interpretation, and manuscript revision. MZ was involved in study conception and design, data acquisition, and manuscript revision. All authors read and approved the final version of the manuscript.

## Appendix

### Nurse-physician relationships items of the Nursing Work Index

1. Physicians and nurses have good working relationships.

2. There is a lot of teamwork between nurses and physicians.

3. There is collaboration between nurses and doctors.

## Pre-publication history

The pre-publication history for this paper can be accessed here:

http://www.biomedcentral.com/1472-6963/10/83/prepub
